# Gambogic Acid Inhibits Gastric Cancer Cell Proliferation through Necroptosis

**DOI:** 10.1155/2023/7532367

**Published:** 2023-08-08

**Authors:** Shujun Wang, Yiping Wang, Hui Zhu, Miaohui Chen, Liang Zhang

**Affiliations:** Department of Gastroenterology, Affiliated Cixi People's Hospital, Wenzhou Medical University, Ningbo, Zhejiang 315300, China

## Abstract

Gambogic acid (GA) is a natural xanthonoid secreted by *Garcinia hanburyi* tree. It possesses anti-cancer activity in various types of cancers. In gastric cancer, it inhibits cell proliferation through increasing apoptosis. However, whether necroptosis is involved in the GA-induced proliferation inhibited in gastric cancer is unknown. In the present study, we found that RIPK1 specific inhibitor necrostatin-1 (Nec-1) attenuated GA-induced proliferation inhibition. GA treatment increased the phosphorylation of necroptosis-related proteins, RIPK1, RIPK3, and MLKL, and their interactions to form the necrosome complex. The effector protein Drp-1 was dephosphorylated by GA treatment. Inhibition of necroptosis by different inhibitors and PGAM5 knockdown attenuated GA-induced cell death in gastric cancer cell lines, thereby attenuating GA-caused cell proliferation inhibition. All the data supported the conclusion that GA could inhibit gastric cancer cell proliferation by inducing necroptosis.

## 1. Introduction

Gambogic acid (GA) is a naturally existing xanthonoid that is presented in the resin secreted by *Garcinia hanburyi* tree [1]. It has been widely investigated for its therapeutic potential in various types of cancers, such as breast cancer, prostate cancer, lung cancer, pancreatic cancer, osteoblastoma, and gastric cancer [1]. GA plays its anti-cancer role through different mechanisms. For example, GA inhibits cancer cell proliferation in melanoma by inducing p66SHC/ROS-p53/Bax mediated apoptosis [2]. In pancreatic cancer, GA treatment could induce autophagy and work with chloroquine to inhibit cancer growth by accumulating reactive oxygen species (ROS) [3]. The biological processes involved in the anti-cancer activity of GA include apoptosis, autophagy, cell cycle arrest, inhibition of cancer cell invasion, metastasis, and angiogenesis [1], but the whole picture of the underlying mechanisms for GA to inhibit cancer progression remains elusive.

Necroptosis is a type of caspase-independent, programmed cell death that has been found to be involved in both pathological and physiological processes [4]. Both intrinsic, such as accumulation of reactive oxygen species (ROS), and extrinsic factors, such as activation of toll-like receptors and TNF receptor superfamily, could induce necroptosis in cells [5, 6]. Necrosome complex, a protein complex formed by receptor-interacting protein kinases 1 and 3 (RIPK1 and RIPK3) and mixed-lineage kinase domain-like pseudokinase (MLKL), mediated the process [4]. In addition, the mitochondrial proteins PGAM5 and Drp-1 function as the effector proteins of necroptosis to induce mitochondria fragmentation [5].

Gastric cancer (GC) ranks fifth in terms of incidence among all cancer types all over the world [7]. Despite the advancement of surgical and chemo-radiotherapy, the morbidity and mortality in GC remain high. There is an unmet need for new therapies to improve the treatment outcome of GC. Several studies on the function of GA in GC have been reported. For example, GA worked as an inhibitor of survival and was able to reverse the drug resistance to docetaxel in gastric cancer cells [8]. In gastric cancer cell line BGC-923, GA treatment induced cell apoptosis to suppress tumor growth [9].

The concept has been established that abnormal cell proliferation and cell death are hallmarks of cancer. Resistance to apoptosis induced by chemotherapeutic agents is often seen in cancer cells. Therefore, induction of necroptosis could be an alternative strategy to kill cancer cells [7]. In fact, dysregulation of necroptosis-related proteins has been found in certain cancer types [10, 11]. In gastric cancer, it has been found that celastrol could induce necroptosis, thereby inhibiting tumor growth [12], suggesting the role of necroptosis in gastric cancer treatment. In addition, GA has been reported to play an inhibitory role in gastric cancer cell survival [5], as well as tumor growth [6]. Although apoptosis has been suggested to be one of the underlying mechanisms for GA-medicated cell death in gastric cancer, other functions of GA in gastric cancer that cause tumor growth inhibition have not been well established. Interestingly, it has been reported that GA treatment could induce the accumulation of reactive oxygen species (ROS) [3], a condition that is able to activate necroptosis, as well as disrupt the TNF signaling pathway, a signaling pathway that is involved in the regulation of necroptosis [13]. Based on these findings about the GA functions in gastric cancer, we hypothesize necroptosis is involved in GA-induced tumor growth inhibition in gastric cancer.

To verify our hypothesis, we treated two different gastric cancer cell lines, AGS and HGC27, with GA or a combination of GA and Nec-1, a specific inhibitor of necroptosis, to see if Nec-1 treatment could rescue cells from GA-induced cancer cell growth inhibition. The results demonstrated that Nec-1 partially rescued the GA-induced inhibition of cancer cell proliferation. Further investigation revealed that GA treatment activated the key regulators of necroptosis, RIPK1, RIPK3, and MLKL, to form the necrosome complex, and the downstream effector proteins of necroptosis, PGAM5 and Drp-1, were also activated. Inhibition of the downstream effector proteins inhibited GA-induced cell death and the anti-cancer activity of GA. All the data suggested that necroptosis is one of the underlying mechanisms of the inhibitory effect of GA in gastric cancer.

## 2. Materials and Methods

### 2.1. Reagents

GA was purchased from Sigma Aldrich (USA) and dissolved in PRMI-1640 medium. Necrostatin-1 (Nec-1) was purchased from Sellecachem (USA). Mdivi-1 was procured from Sigma Aldrich (USA). Necrosulfonamide (NSA) was procured from Sigma Aldrich (USA). The antibodies used in the present study included anti-phospho-RIPK1 (Ser166) (Cell Signaling Technology, USA), RIPK1 (Cell Signaling Technology, USA), anti-phospho-RIPK3 (Ser227) (Abcam, USA), RIPK3 (Cell Signaling Technology, USA), anti-phospho-MLKL (Ser358) (Abcam, USA), anti-MLKL (Sigma, USA), anti-PGAM5 (Abcam, USA), anti-phospho-Drp-1 (S637) (Cell Signaling Technology, USA), anti-Drp-1 (Santa Cruz, USA), and anti-GAPDH (Santa Cruz, USA).

### 2.2. Cell Culture

Gastric cancer cells AGS and HGC-27 were cultured in RPMI-1640 medium containing 10% fetal bovine serum (FBS; Thermo Fisher Scientific, MA, USA) and 1% penicillin-streptomycin in an incubator with 5% CO_2_ at 37°C. AGS is a gastric cancer cell line derived from gastric adenocarcinoma. HGC27 cell is a gastric cancer cell line derived from the lymph node metastasis tissues of a patient diagnosed as undifferentiated carcinoma.

### 2.3. 3-(4,5-Dimethylthiazol-2-Yl)-2,5-diphenyltetrazolium Bromide Assay (MTT Assay)

AGS or HGC-27 cells were seeded in a 96-well plate at the density of 1 × 10^4^ per well, respectively, and cultured overnight. The cells were then washed with PBS once and cultured with culture medium, culture medium containing 2 *μ*M GA, or culture medium containing indicated inhibitors. The treatment concentrations of the inhibitors were 2 *µ*M for GA, 20 *μ*M for Nec-1, 2 *μ*M for NSA, and 50 *μ*M for Mdivi-1 as they have been proved effective in other publications [9]. Cells were treated for 24, 48, and 72 hours, respectively. After incubation, 10 *µ*l MTT solution (5 mg/ml) was added to each well and the cells were cultured at 37°C for additional 4 hours. Then, the cells were lysed in 100 *μ*l DMSO and the whole plate was read by a microplate reader at OD 490 nm. Each treatment condition was triplicated. The experiment was repeated independently three times.

### 2.4. Colony Formation

AGS and HGC27 cells were seeded in a 6-well plate at the density of 5 × 10^3^/well and cultured overnight. The cells were washed with PBS once, and the culture medium was refreshed using the culture medium containing indicated inhibitors. Complete medium without GA or any inhibitors was used as control. The cells were then cultured in an incubator with 5% CO_2_ at 37°C for 2 weeks. The culture medium was refreshed every 3 days. The concentrations of GA, Nec-1, NSA, and Mdivi-1 were 2 *μ*M, 20 *μ*M, 2 *μ*M, and 50 *μ*M, respectively. After 2 weeks, the culture medium was gently removed, and the cells were washed with PBS once, followed by fixation with 10% neutral buffered formalin for 20 minutes. After the cells were fixed, the formalin solution was gently removed, and the colonies were stained with 0.01% (w/v) crystal violet in dH_2_O for 30 minutes followed by washing with excess water. The plate was let in a laminar hood for air drying, and the colonies with a diameter no smaller than 1 mm were counted. Samples for each experiment condition were triplicated. Also, the experiment was repeated independently 3 times.

### 2.5. Annexin-V/Propidium Iodide (PI) Staining of Cells

A cell death detection kit from Abcam (ab14085) was used to stain the cells with annexin V-FITC and propidium iodide (PI). A coverslip was placed in each well of a 6-well plate, and 5 × 10^5^ cells were seeded in each well to grow the cells on the coverslip and cultured overnight. On day 2, indicated chemical agents were added to the culture medium for 24 hours. The cells were then gently washed with PBS and incubated in 1 × binding buffer. Annexin-V-FITC and PI were added into the binding buffer at 1 : 100 ratio and incubated with cells for 5 minutes in the dark. After staining, cells were gently washed with PBS and fixed with 2% paraformaldehyde. The coverslip was inverted and placed onto a glass slide and observed under a fluorescent microscope. At least 3 randomly selected fields were counted for annexin/PI double staining cells.

### 2.6. siRNA Transfection and Stable Cell Line Generation

PGAM5 siRNA was synthesized and purchased from Sangon Biotech (China). The sequence was CGGAAGCTGTGCAGTATTA. Cells were seeded in a 6-well plate at the density of 4 × 10^4^/well and cultured overnight. Then, the cells were transfected with 200 pmol siRNA in each well and further cultured for 3 days. At the end of day 3, cells were harvested for further investigation.

The same siRNA sequence was used to construct a PGAM5 shRNA expressing vector. The plasmid was used to generate a PGAM5 knockdown cell line in both AGS and HGC27 cell lines by puromycin selection.

### 2.7. Cell Lysate Preparation

For cell lysate used for coimmunoprecipitation assay, cells were lysed following the protocol described previously after being treated with indicated agents [7]. In brief, cells were washed twice with ice-cold PBS before being lysed in the lysis buffer (20 mM Hepes, pH 7.4, 1% TritonX-100, 40 mM KCl, 1.5 mM MgCl_2_, 1 mM EDTA, 1 mM EGTA, 0.1 mM PMSF, and 250 mM sucrose). Then, 250 *μ*l ice-cold cell lysis buffer was added to each well in the six-well plate. The cell lysate was incubated on ice for 30 minutes followed by homogenization by passing through a 22-G needle 24 times. After homogenization, the lysate was centrifuged at 10000*g* for 10 minutes. The supernatant was removed to a new tube and centrifuged again at 15000*g* for another 10 minutes. The supernatant was removed to a new tube and saved for coimmunoprecipitation.

For total cell lysate preparation, cells were washed with PBS twice before being lysed in the lysis buffer described above with 0.1% SDS (20 mM Hepes, pH 7.4, 1% TritonX-100, 40 mM KCl, 1.5 mM MgCl_2_, 1 mM EDTA, 1 mM EGTA, 0.1 mM PMSF, and 250 mM sucrose). The procedure to prepare the total cell lysate was the same as the protocol to prepare cell lysis for coimmunoprecipitation.

### 2.8. Western Blot Analysis

For western blot analysis, 30 *μ*g total cell lysate samples were resolved by 4–20% gradient SDS-PAGE gel. After electrophoresis, the proteins were transferred to PVDF membrane and blocked with 5% non-fat milk in PBS for 1 hour at room temperature. Then, the membrane was incubated with indicated primary antibodies overnight at 4°C. The primary antibodies were diluted at 1 : 1000 in 5% non-fat milk. After incubation, the membrane was rinsed with 5% non-fat milk in PBS 3 times, 10 minutes each time, followed by incubation with secondary antibody diluted at 1 : 5000 at room temperature for 1 hour. Then, the membrane was washed three times, 10 minutes each time, followed by rinsing with PBS-T (0.05% Triton X-100) for 5 minutes. The membrane was briefly air-dried, and the enhanced chemiluminescence reagent (Amersham ECL, Amersham, United Kingdom) was used to visualize the results by following the manufacturer's instruction.

### 2.9. Coimmunoprecipitation (Co-IP)

The cell lysate was prepared as described in the cell lysate preparation section, and an equal amount of cell lysate (1 mg) was used for immunoprecipitation assay. 5 *μ*g anti-RIPK3 antibody for each sample was used to incubate with cell lysate at 4°C overnight by gently inverting the sample tube. Then, 10 *µ*l protein A or protein G conjugated agarose beads were added to the sample depending on the primary antibody used for Co-IP and further incubated with the sample for 1 hour at room temperature by gently inverting the tube. The beads were then pelleted by a centrifuge at 2500*g* for 3 minutes and washed with lysis buffer for 3 times. After 3 times of washing, the beads were pelleted, 50 *µ*l 1 × SDS loading buffer was added to each tube to resuspend the beads, and the samples were boiled for 5 minutes in a water bath. The samples were then centrifuged at 12000*g* for 2 minutes, and the supernatant was loaded to and resolved in 4–20% SDS-PAGE gel for electrophoresis. Western blot was used to visualize the interactions among the protein complex.

### 2.10. Statistics

All data were analyzed with the software GraphPad Prism (GraphPad Software Inc, San Diego, CA, USA), and presented as mean ± SD from at least 3 independent experiments. We applied Student's *T*-test with two tails to compare the difference between 2 groups assuming unequal variance. *p* < 0.05 was considered statistically significant.

## 3. Results

### 3.1. Nec-1 Partially Rescued Gastric Cancer Cells from GA-Induced Cell Proliferation Inhibition

**Figure 1:**
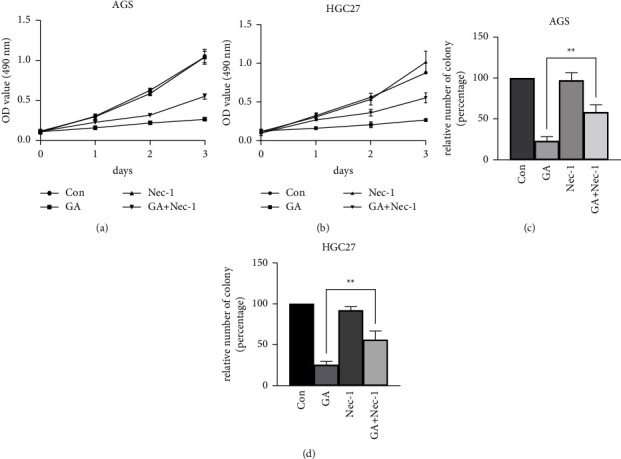
Nec-1 attenuated GA-induced proliferation inhibition. 2 *µ*mol/L GA and 20 *µ*mol/L Nec-1 were used to treat cells for 3 days, and MTT assay was performed to measure viable cells in AGS (a) and HGC27 (b) cells at the indicated time point. (c) and (d), cells were treated with GA and Nec-1 with the same concentration in (a) and (b), and colony formation assay was performed to test the cell proliferation. (c) AGS cells. (d) HGC27 cells.

It has been reported that GA could inhibit gastric cancer cell proliferation at a range of concentrations [6]. In HGC27 and AGS cells, 2 *μ*M GA could effectively inhibit cell proliferation [14]. Therefore, 2 *μ*M GA was used in gastric cancer cell treatment in the present study. We first employed MTT assay to test the effect of GA on cell proliferation. As shown in Figures 1(a) and 1(b), GA effectively inhibited cell proliferation in both AGS and HGC27 cells, which was consistent with previous results [7]. Next, we tested if Nec-1, a specific inhibitor of RIPK1, could block the GA-induced inhibition of cell proliferation. 20 *μ*M Nec-1 was used to treat the cells for 1 hour, followed by the addition of 2 *μ*M of GA. The cells were cultured in the presence of the two agents for 3 days. The results clearly demonstrated that pretreatment of cells with Nec-1 significantly improved cell proliferation by approximately 2 fold in both AGS and HGC27 cells, suggesting the roles of RIPK1 in GA-mediated cell proliferation inhibition. Next, we employed colony formation assay to test the effect of GA and GA + Nec-1 on tumor cell survival and proliferation. Colony formation assay is a well-established in vitro assay to evaluate the survival and proliferation of cancer cells. It has been widely used and has become a standard method in cancer research to assess cell proliferation and the cytotoxic effects of various agents, which may have the potential as anti-cancer therapeutic agents. These agents include chemotherapeutic agents and targeted therapies, individually or in combination. Results from the colony formation assay were consistent with the results from the MTT assay. As shown in Figures 1(c) and 1(d), GA treatment greatly reduced the number of colonies in AGS and HGC27 cells. The number of colonies in the GA-only treated group was about 19% and 22% of the control group in AGS cells and HGC27 cells, respectively. Upon the addition of Nec-1, the decrease in colony numbers was partially reversed compared to the GA-only group. The number of colonies increased to about 54% and 50% of the control group in AGS and HGC27 cells, respectively. The difference between the GA-only and the GA + Nec-1 treated groups was statistically significant. This result suggested that Nec-1 treatment partially rescued the GA-induced cell death and cell proliferation inhibition.

### 3.2. Nec-1 Ameliorated GA-Induced Cell Death in Gastric Cancer Cells

**Figure 2:**
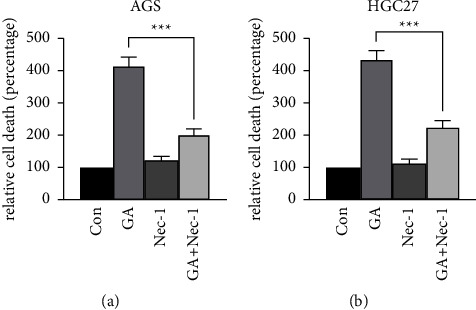
Nec-1 treatment partially blocked GA-induced cell death. Cells were treated with GA and Nec-1 for 24 hours. Annexin V/PI staining was used to stain the cells to identify apoptotic and necroptotic cells. Annexin V-FITC^+^/PI^+^ cells were considered apoptotic/necroptotic cells at the late stage. (a) AGS cells. (b) HGC27 cells. At least 3 randomly selected fields under the microscope were counted for each sample.

GA has been known for its anti-cancer activity through enhancing cell apoptosis [6]. Nec-1 is a specific inhibitor of RIPK1. RIPK1 is an important regulator of another type of programmed cell death, necroptosis. We wonder if Nec-1 blocked GA-induced proliferation inhibition by ameliorating GA-induced cell death. After treatments, annexin-V and PI staining were used to detect cell death. As shown in Figure 2, the number of annexin-V/PI double-stained cells in the GA-only treated cells increased to about 4 fold compared to that of control cells, while the number of double-stained cells decreased by roughly 50% compared to the GA-only treated cells. The difference between the GA-only treated group and the GA + Nec-1 treated cells was statistically significant (Figures 2(a) and 2(b)). The result was consistent in both AGS and HGC27 cell lines, suggesting that necroptosis plays a role in GA-induced cell death in gastric cancer.

### 3.3. GA Activated Necroptosis through RIPK1/RIPK3/MLKL/PGAM5/Drp-1 Pathway

**Figure 3:**
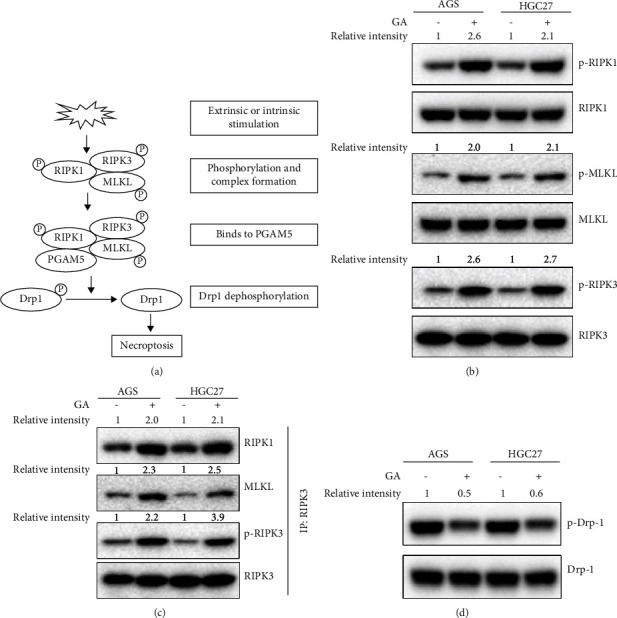
GA treatment activated necroptosis signaling pathway in gastric cancer cells. AGS and HGC27 cells were treated with 2 *µ*mol/L GA for 24 hours. The treated cells were washed and harvested in lysis buffer. Western blot was used to detect the phosphorylation of each indicated necroptosis-related protein. (a) Schematic cartoon showing activation of necroptosis. (b) Phosphorylation of indicated necroptosis-associated proteins. (c) Coimmunoprecipitation assay. 1 mg total cell lysate of each indicated sample was used to perform the coimmunoprecipitation assay. 5 *μ*g RIPK3 antibody for each sample was used to do the assay. RIPK1, RIPK3, phospho-RIPK3, and MLKL were detected by western blot with corresponding antibodies. (d) Phosphorylation of Drp-1.

We then examined if GA treatment activated the RIPK1 mediated necroptosis signaling pathway. In brief, upon activation of necroptosis activation, RIPK1, RIPK3, and MLKL will be phosphorylated and form a complex. The protein complex will then bind to mitochondrial protein phosphoglycerate mutase/protein phosphatase (PGAM5), which in turn will dephosphorylate the downstream effector protein Drp-1 to activate its GTPase activity [5], thereby causing mitochondrial fragmentation and cell death. A schematic figure to show necroptosis pathway is shown in Figure 3(a). To test if necroptosis signaling pathway was involved in the GA-induced anti-cancer activity in gastric cancer, AGS and HGC27 cells were treated by GA for 24 hours, and the cells were harvested for the analysis of the necroptosis pathway activation. The total cell lysate of untreated and GA-treated AGS and HGC27 cells was resolved by SDS-PAGE electrophoresis, and antibodies specifically binding to the corresponding phosphoproteins were used to determine the phosphorylation status of key signaling factors in the necroptosis pathway. As shown in Figure 3(b), upon GA treatment, the phosphorylation of RIPK1, PIPK3, and MLKL was increased to 2-3 fold compared to the untreated cells, suggesting the activation of the signaling pathway upon GA treatment. The necrosome complex was also formed (Figure 3(c)). The interactions among RIPK1, MLKL, and phosphorylated PRIK3 were enhanced upon GA treatment to the level of roughly 2 fold as in the untreated cells. Drp-1 protein functions as the ultimate effector to cause mitochondria fragmentation in the necroptosis pathway. Dephosphorylation of Drp-1 at serine 637 indicates its activation. Thus, we checked the activity of Drp-1 by detecting its phosphorylation status. As shown in Figure 3(d), Drp-1 phosphorylation decreased to 50–60% in GA-treated cells compared to that in the untreated cells, in both AGS and HGC27 cell lines. All the results suggested that GA treatment activated RIPK1/RIPK3/MLKL/PGAM5/Drp-1 medicated necroptosis pathway.

### 3.4. Inhibition of Necroptosis Suppressed GA-Induced Cell Death

**Figure 4:**
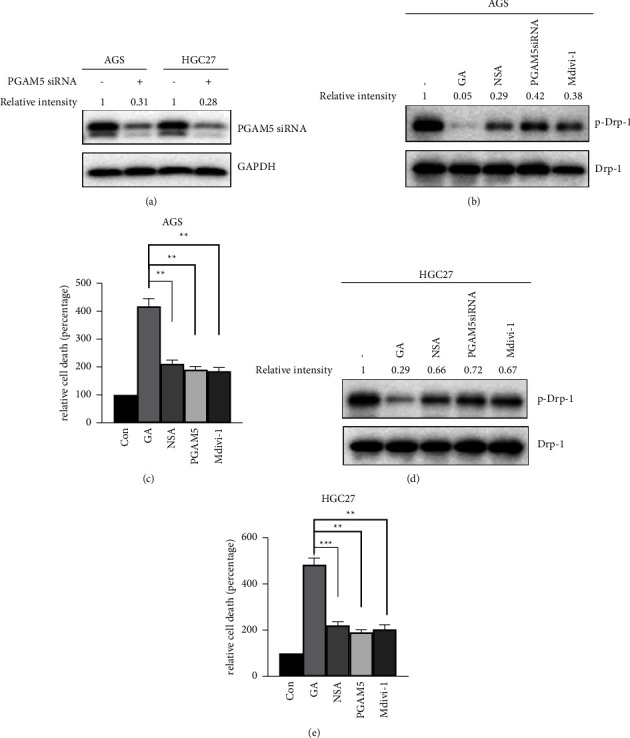
Necroptosis inhibitors inhibited GA-induced cell death. Indicated inhibitors were used to treat cells to inhibit necroptosis at a different step. Knockdown of PGAM5 was used to attenuate PGAM5 activity. (a) PGAM5 expression was checked by western blot in PGAM5 siRNA transfected cells. (b) Inhibitors were used to treat AGS cells for 24 hours and the phosphorylation of Drp-1 was examined by western blot in treated cells and PGAM5 siRNA transfected cells. (c) Annexin V-FITC/PI double staining of AGS cells treated with GA and indicated inhibitors or PGAM5 knockdown to detect cell death. (d) Inhibitors were used to treat AGS cells for 24 hours and the phosphorylation of Drp-1 was examined by western blot in treated cells and PGAM5 siRNA transfected cells. (e) Annexin V-FITC/PI double staining of AGS cells treated with GA and indicated inhibitors or PGAM5 knockdown to detect cell death. ^*∗∗*^*p* < 0.01; ^*∗∗∗*^*p* < 0.001.

Next, we tested if necroptosis was involved in GA-induced cell death in gastric cancer by employing different inhibitors of necroptosis at various steps. Necrosulfonamide (NSA) is a specific inhibitor for MLKL. Mdivi-1 specifically inhibits Drp-1 activation. For PGAM5, we established a stale cell with PGAM5 shRNA to reduce its expression level.  Figure 4(a) shows the decrease of PGAM5 expression in both AGS and HGC27 PGAM5 knockdown cell lines. As shown in the figure, the expression of PGAM5 decreased to about one-third of the expression level in control siRNA transfected cells (Figure 4(a)). As shown in Figure 4(b), cells were mock treated (-), 2 *μ*M GA treated (GA), 2 *μ*M NSA + 2 *μ*M GA treated (NSA), or 50 *μ*M Mdivi-1 + 2 *μ*M GA treated for 24 hours, the cells were harvested, and the total cell lysate was used to detect the phosphorylation status of Drp-1, as phosphorylation of Drp-1 was negatively correlative with the necroptotic activity in cells. Dephosphorylation of Drp-1 at serine 637 indicates the activation of the protein [13]. As shown in Figure 4(b), either treatment with inhibitors or PGAM5 knockdown partially rescued the dephosphorylation of Drp-1 caused by GA treatment in AGS cells. Consistently, the cell death induced by GA treatment was inhibited by the indicated inhibitors or PGAM5 knockdown (Figure 4(c)), and the cell death induced by GA treatment was inhibited to about 50% by the indicated inhibitors or PGAM5 knockdown. The difference between the GA + inhibitor treated cells and the GA-only treated cells was statistically significant. Similar results were also found in HGC27 cells, which are shown in Figures 4(d) and 4(e). All the data demonstrated that GA treatment activated necroptosis signaling pathway and induced cell deaths.

### 3.5. Inhibition of Necroptosis at Different Steps Partially Reversed GA-Induced Cell Proliferation Inhibition

**Figure 5:**
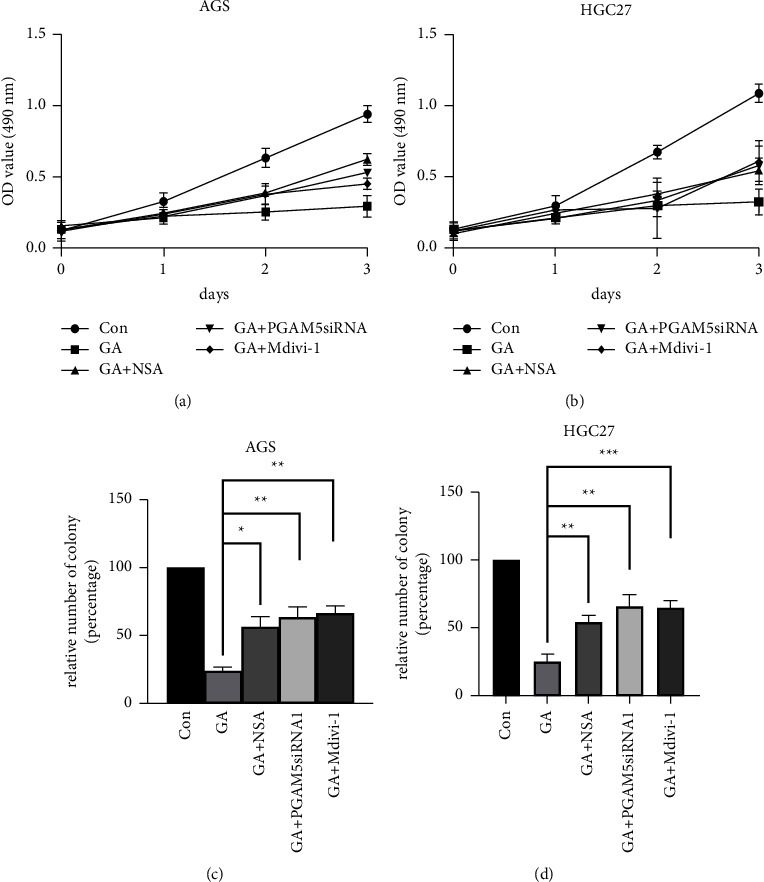
Necroptosis inhibitors ameliorated GA-induced cell proliferation inhibition. AGS cells or HGC27 cells were treated with indicated inhibitors as described in Materials and Methods. PGAM5 knockdown stable cell line was also used. MTT assay ((a) AGS; (b) HGC27) and colony formation assay ((c) AGS; (d) HGC27) were performed to test the proliferation of the cells after treatment. ^*∗*^*p* < 0.05; ^*∗∗*^*p* < 0.01; ^*∗∗∗*^*p* < 0.001.

All the above data demonstrated that GA treatment activated necroptosis in gastric cancer cells. Next, we tested if necroptosis mediated GA-induced cell proliferation inhibition. MTT assay and colony formation assay were employed to test the effect of indicated necroptosis inhibitors and the PGAM5 knockdown on cell proliferation in both AGS and HGC27 cell lines. As described in the Materials and Methods section, cells were seeded, mock treated, treated with GA only, or treated with indicated inhibitors in combination with GA. As shown in Figure 5, inhibitor treatment and PGAM5 knockdown significantly rescued cells from GA-induced proliferation inhibition, which was demonstrated by the lower OD value reading in the MTT assay or the smaller number of colonies in the colony formation assay. The difference between GA-only treated cells and the inhibitor + GA treated cells was statistically different, and the data were consistent in AGS cells (Figures 5(a) and 5(c)) and HGC27 cells (Figures 5(b) and 5(d)).

## 4. Discussion

In the present study, we investigated the role of necroptosis in GA-induced inhibition of cell proliferation in gastric cancer cell lines. The results demonstrated that GA treatment activated the necroptosis signaling pathway, which was shown by the increased phosphorylation of RIPK1, RIPK3, and MLKL, formation of necrosome complex by the three proteins, and the dephosphorylation of Drp-1, the downstream effector protein of necroptosis. Inhibition of necroptosis by different inhibitors reduced phosphorylation of key regulators of necroptosis, deactivated Drp-1, and ameliorated GA-induced cell death, thereby inhibiting GA-induced proliferation inhibition. To our best knowledge, this is the first study about the role of necroptosis in the anti-cancer activity of GA in cancer.

Targeting necroptosis may be an alternative strategy to treat cancers because a lot of chemotherapy treatments could possibly induce resistance to apoptosis-mediated cell death in cancer [7], which is also true in gastric cancer. The roles of necroptosis in tumorigenesis in different types of cancers may vary due to the origin of the cancer cells. A recent study reported abnormal expression patterns of the proteins in the necroptosis signaling pathway in gastric adenocarcinoma [15], indicating that necroptosis was involved in the tumorigenesis and development of gastric cancer. The Epstein–Barr virus, an infective agent that could cause gastric cancer, encodes non-coding RNAi that could regulate the expression of necroptosis-related proteins in infected gastric cancer cells. It eliminates MLKL and reduces the expression of RIPK1 via different non-coding RNAs the virus encodes [16]. Targeting necroptosis might be a new strategy for gastric cancer treatment. In fact, results from several recently published studies provided evidence supporting this hypothesis. Astaxanthin treatment could induce the activation of NADPH oxidase and the activation of RIPK1 medicated necroptosis in a gastric cancer cell line named AGS [17]. Celastrol, a compound extracted from traditional Chinese herbal medicine, induced necroptosis and reduced inflammation by targeting bi-glycan pathways [12].

It has been reported that GA induces apoptosis to inhibit cell proliferation in gastric cancer cell line BGC-823 [6]. However, it does not necessarily exclude the role of necroptosis in GA-mediated anti-cancer activity. The exact mechanism underlying the activation of necroptosis by GA treatment in gastric cancer cells remains unclear. Nonetheless, we proposed several hypotheses. First, it may cause the activation of necroptosis through an extrinsic pathway, possibly upon activation of the TNF receptor superfamily. It has been reported that GA could interact with the transferrin receptor and potentiate TNF-induced apoptosis through inhibition of the nuclear factor-*κ*B signaling pathway [13], suggesting that GA treatment activates TNF pathway. TNF receptor-mediated signaling pathway is a typical extrinsic signal that leads to necroptosis [7]. NF-*κ*B was an important downstream effector of TNF signaling pathway. Inhibition of NF-*κ*B signaling pathway may shift the TNF-activated signaling events to anti-survival events, including apoptosis and necroptosis. Second, GA treatment could increase the concentration of ROS in cells [6, 18]. ROS accumulation in cells could induce necroptosis. Mitochondrial ROS was reported to be responsible for the autophosphorylation of RIPK1 and the recruitment of RIPK3 to necrosome [19]. Thus, the ROS accumulation in cells induced by GA treatment is one of the possible explanations for necroptosis activation by GA. Further experiments are required to verify these hypotheses.

The application of GA in combination with other chemotherapeutic agents may augment the treatment effect since GA would target necroptosis in addition to apoptosis. In pancreatic cancer cells, GA induced autophagy and worked with chloroquine to synergistically inhibit cell proliferation [3]. A number of chemotherapeutic agents exert their anti-cancer activities by promoting apoptosis [20]. Combinational treatment of these commonly used chemotherapeutic agents with GA may synergistically improve the treatment outcome.

In addition to synergistically suppress tumor cell growth with other chemotherapeutic agents, GA may also exert its anti-cancer activity by inhibiting tumor angiogenesis, a critical process for tumor growth. The concept has been proven in prostate cancer [21]. GA treatment inhibited human umbilical vein endothelial cell (HUVEC) proliferation, migration, and tube formation [21]. In a xenograft model, GA treatment demonstrated a better inhibition on HUVEC cells than prostate cancer cells, suggesting the potential of GA as an anti-angiogenesis agent. Gambogic amide, a derivative of GA, has also been shown to inhibit angiogenesis, expression of VEGF, and the activation of VEGF/VEGFR2 signaling in HUVEC cells and normal human endothelial cells (NhEC) [22]. All the study results indicate the role of GA in angiogenesis in GC. Angiogenesis plays an important role in tumor malignancy and progression. In fact, there are excellent reviews about the angiogenesis, crosstalk between vascular cells and the immune cells in blood, and role of endothelial cells for immunological patrolling in the microenvironment of a tumor mass [23, 24]. The blood vessels formed in the tumor lack normalcy and interact with immune cells in the blood stream, creating an immunosuppressive environment in tumor mass with the presence of tumor cells. Therapies targeting anti-angiogenesis or targeting immune check points in tumor environment have been developed to treat various tumors, such as anti-VEGF antibody, bevacizumab, and anti-PD-1 antibody, pembrolizumab.

Combinational treatment of GA with other agents targeting malignancies other than necroptosis/apoptosis in tumor, such as angiogenesis and suppressive immune environment, is one of the next directions for GA studies in cancer. As indicated in the previous studies, GA treatment may not only kill tumor cells but also inhibit abnormal angiogenesis in tumor, thereby inhibiting nutrient supply to tumor. Combination of GA with anti-angiogenesis agents may achieve a synergistic response.

Although there is a great potential benefit of GA treatment in gastric cancer, one important aspect that needs to be considered is the toxicity of the compound, both as a single agent or in a combinational treatment. Systemic use of GA may induce systemic reactions to the compound which is not desired by the drug developer. How to achieve a desired efficacy yet avoiding intolerable toxicity is a key question to resolve for all new drug development, including GA. In the current study, such issue is not investigated or discussed, which is a limitation of the current study, but it will be one of the directions to explore in the future to further understand the role of GA in GC treatment. Target therapy, which may couple GA to a targeting vehicle specifically binds to the biomarker which is only or exclusively located in the tumor. It may also help achieve the goa. Further investigation is required to explore this direction.

## Data Availability

The data used to support the findings of this study are available from the corresponding author upon request.
